# Comparison of Ultrasound- and Microwave-Assisted Extraction Techniques on Chemical, Technological, Rheological, and Microstructural Properties of Starch from Mango Kernel

**DOI:** 10.3390/gels11050330

**Published:** 2025-04-29

**Authors:** Luis Mieles-Gómez, Somaris E. Quintana, Luis A. García-Zapateiro

**Affiliations:** Research Group on Complex Fluid Engineering and Food Rheology, Universidad de Cartagena, Cartagena 130015, Colombia; lmielesg@unicartagena.edu.co (L.M.-G.); squintanam@unicartagena.edu.co (S.E.Q.)

**Keywords:** starch, ultrasound-assisted extraction, microwave-assisted extraction, technofunctional properties, rheological properties

## Abstract

The effect of emergent technologies for the starch extraction was studied. The evaluation of conventional extraction (MKS-WMP), ultrasound-assisted extraction (MKS-UAE), and microwave-assisted extraction (MKS-MAE) on chemical, technological, gelling, pasting, and microstructural properties of starch from mango kernel was carried out. The extraction yield was found in the values of 42.05, 50.40, and 47.43% for MKS-WMP, MKS-UAE, and MKS-MAE treatments, respectively. The amylose contents for MKS-UAE and MKS-MAE starches were significantly higher (*p* < 0.05) than MKS-WMP, with an increase of about 13–18%. The total phenolic content ranged from 84.89 to 90.85 mg GAE/g starch without significant differences (*p* > 0.05). The technological properties evidence a good water-holding capacity (80.48–90.05 g/100 g of starch) and oil-holding capacity (70.58–83.23 g/100 g of starch). The gelatinization temperature, measured by rheological analysis, ranged between 77 and 82 °C. Microstructural properties showed that ultrasound- and microwave-assisted treatments improved the shape and surface of starch granules, and that they are promising alternatives for starch extraction, providing some characteristics that could increase the applications in the food industry.

## 1. Introduction

Mango (*Mangifera indica*) is among the most widely traded tropical fruits globally, ranking second in commercial exchange and fifth in production volume. Recognized as the ‘king of fruits’, it is consumed at different ripening stages due to its distinctive and appealing flavor [1]. In addition, mango consumption is associated with numerous health benefits due to its composition, including phytochemicals and a rich nutritional profile [2]. Over 50% of the mangoes harvested are processed into various products, such as juice, nectar, puree, jam, and pickles, among others [3]. This generates large quantities of by-products, primarily composed of peels and seeds, which pose environmental challenges for both industry and local communities [4]. The edible portion of the mango fruit varies depending on the variety and accounts for 33–85% of the fresh weight; the seed represents 20–60% of the total fruit mass, of which the kernel comprises 45–75%. The main component of the mango seed kernel is starch, ranging from 38 to 70% [5], which makes it an interesting polysaccharide that can be used from the seed.

Among plant-derived polysaccharides, starch is ranked as the second most abundant after cellulose and plays a key role as an energy source in both food systems and biopolymer applications [6]. It has varied applications in foods (additives such as stabilizers, thickeners, gelling agents, edible films, and encapsulating compounds) and in non-food sectors, including the paper, textile, and cosmetic industries. It is primarily extracted from conventional sources, including cereals and tubers, thus driving demand along the supply chain of these food groups while also impacting costs and environmental sustainability [7]. However, in recent times, a wide variety of new sources of starches have been found that represent an alternative to native starches associated with technological properties similar to or better than those of commercial starches [5]. Various studies have indicated that starch derived from mango seeds exhibits significant potential to compete with commercially relevant starches, such as those from corn, potatoes, and beans, in industrial applications [8,9].

However, to understand and propose an adequate technological application of mango seed starches, it is necessary to explore different extraction techniques to obtain starches with a higher yield rate, greater purity, and desirable functional properties. In this sense, ultrasonic treatment emerges as an effective alternative starch extraction technique. Ultrasonic waves generate cavitation phenomena in liquid media, creating microbubbles that collapse violently and release localized energy. This process disrupts cell walls and facilitates the release of intracellular compounds, such as starch. In starch extraction, ultrasound primarily affects the amorphous regions of the granules, leading to surface erosion, increased porosity, and improved solvent penetration [10,11]. These effects can enhance extraction yield and modify functional properties such as solubility, swelling power, and gelatinization behavior. In addition, it allows for lower consumption of chemical products and shorter extraction times. It also offers great potential for industrial application due to its flexibility to integrate with other processes, low cost, high efficiency, and environmentally friendly profile [10,11]. Likewise, microwaves are non-ionizing electromagnetic waves that generate molecular micromovements and friction in food through high-frequency electric fields, operating based on the dielectric loss principle. Microwave processing can break glycosidic bonds, promote the structural reorganization of starch molecules, and alter the morphology and relative crystallinity of starch granules, leading to modifications in paste viscosity, swelling ability, emulsifying stability, and emulsification properties [12]. Therefore, microwave treatment has gained immense commercial and industrial importance over the years and is being explored for its compatibility with different sources.

Different researchers have used ultrasound and microwave-assisted technology to isolate starch from different botanical sources, such as some legumes and roots [13,14,15]. Despite this potential, little research has focused on comparing their effect on the technological, rheological, and microstructural properties of mango kernel starch. Consequently, the main objective of this research was to evaluate the effect of ultrasound- and microwave-assisted extraction techniques on mango kernel starch, analyzing their chemical, techno-functional, gelling, pasting, and microstructural properties.

## 2. Results and Discussion

Mango seed starch (MKS) was obtained using conventional wet-milling process (MKS-WMP), ultrasonic-assisted extraction (MKS-UAE), and microwave-assisted extraction (MKS-MAE), obtaining three starch samples with similar characteristics, that is, a powder with a soft texture and light brown color.

The extraction yield was found in the values of 42.05%, 50.40%, and 47.43% for MKS-WMP, MKS-UAE, and MKS-MAE treatments, respectively (Table 1). Then, the yield extraction of starch by wet milling (conventional method) was close to that reported for mango seed starch of Indian [16] and Brazilian [17] origin, obtained via the wet-milling process, presenting values of 39.35 and 44.95%, respectively. Furthermore, regarding the extraction method, the yield of starches depends on other factors, such as the origin and the condition of the fruit, the requirements of the soil, etc. [18]. However, ultrasound and microwave-assisted extraction presented a significant effect (*p* < 0.05) on starch yield compared to the conventional method. The highest performance value was obtained in the UAE treatment, but without significant differences (*p* > 0.05) with the MAE treatment. These results could be related to the dominant effects of ultrasound cavitation and microwave irradiation. The cavitation effect contributes to increasing the mass transfer of intracellular substances to the solvent, because the particles decompose during sonication, which exposes the structure of the fibrous material of the mango seed to release more starch granules to the outside [19], improving the extraction performance. Likewise, during the MAE process, the solutes are in contact with the solvent, so microwave irradiation can improve the solubility of the compounds and their diffusion from the cells to the solvent, because the plant cells expand and are broken due to the absorption of microwaves, and the active substances of the cells, such as starch, are released and dissolved in the solvent [20]. MAE exhibits good efficiency and shorter extraction time than other extraction techniques. Similar results have been found in obtaining polysaccharides from *Panax notoginseng* by ultrasound and microwave-assisted extraction [21], and microwave/ultrasound-assisted extraction of phytocompounds from the pulp of *Syzygium cumini* [22].

### 2.1. Amylose Content

Amylose plays a key role in defining the functional behavior of starch, as it influences gelatinization temperature, retrogradation tendency, gel firmness, and overall textural properties in food systems [23]. The amylose content of starch present values between 28.46 and 33.84 g/100 g of starch, as detailed in Table 1. Then, the amylose contents for MKS-UAE and MKS-MAE starches were significantly higher (*p* < 0.05) than MKS-WMP, with an increase of about 13–18%. These results can be attributed to the partial depolymerization of amylose and amylopectin during ultrasonic extraction, which leads to an increased number of linear chains and, consequently, a higher amylose content in starch granules [24]. Similarly, microwave irradiation causes the polar water molecules to vibrate and breakage of the α-1,4 and α-1,6 glycosidic bonds to occur; therefore, the amylose trapped within the granular structure by the amylopectin groups escapes from the semicrystalline structure [25]. The results obtained are consistent with the report that ultrasonic pretreatment increased the amylose content in corn starch at high concentrations [23] and the report that, compared to conventionally extracted starch and without any modification, starch thermally treated with microwave had a higher amylose content [26].

### 2.2. FTIR Analysis

**Figure 1:**
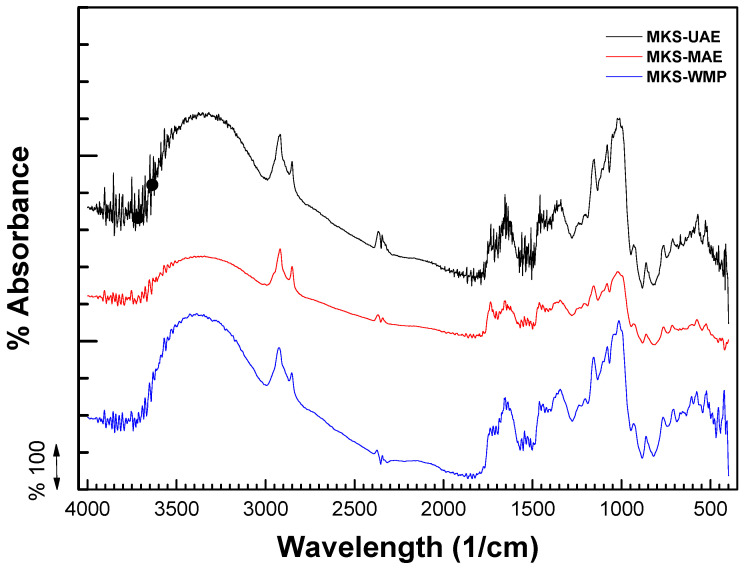
FTIR spectrum of mango kernel starch by wet-milling process (MKS-WMP), ultrasound-assisted extraction (MKS-UAE), and microwave-assisted extraction (MKS-MAE).

FTIR spectroscopy has proven to be an effective technique for identifying functional groups in biopolymers, offering valuable insights into structural features such as double-helical arrangement, helicity, and chain conformation within a limited range [27]. The FTIR spectra in the range from 4000 to 550 cm^−1^ of the conventionally extracted starches (WMP), assisted by ultrasound (UAE) and microwaves (MAE), are shown in Figure 1. The FTIR spectrum reveals characteristic peaks at various wavenumbers, reflecting the distinct structural features of the starches. The spectrum pattern did not change, and there was no appearance or loss of new absorption peaks for all samples, demonstrating that no new functional groups or new chemical bonds were formed after the ultrasound- or microwave-assisted extraction technique. Results agree with those reported for pea starch during the process assisted by pre- and post-ultrasound treatment [24] and millet starch treated under microwaves and ultrasound at the same power [28].

The region at 1200–800 cm^−1^ is the band sensitive to the configuration of starch. For all samples, a strong peak was observed around 1022 cm^−1^; the absorption peak of this part mainly reflects the vibration of C-O, C-C and C-OH bonds and is related to the vibrational absorption of the crystalline regions and starch amorphous. Furthermore, a decrease in the intensity peak was evident in the MKS-MAE sample; this could be attributed to the modification of the starch structure and the development of new intramolecular hydrogen bonds when water evaporates through microwave treatment [29]. Similarly, the peak presented in the wavelength range of 1170–1150 cm^−1^ is the peak of the carbonyl ester, which initially indicates that mango seed starches contain some compounds that are not found in the starch obtained from conventional sources. The peak at 1650 cm^−1^ is related to the bending of water in the amorphous region of starch granules [30]. A broad peak was recorded around 3600–3000 cm^−1^ which represents the stretching vibration of the –OH group of water and starch. The small peak at 2900 cm^−1^ is attributed to C-H stretching of aliphatic groups, reflecting the presence of small amounts of lipids [31].

### 2.3. Total Phenolic Compounds (TPC) and Antioxidant Capacity

The determined TPC and the antioxidant capacity of starches are shown in Table 1. The TPC content of the starch samples ranged from 84.89 to 90.85 mg GAE/g starch without significant differences (*p* > 0.05); that is, the extraction assisted by ultrasound and microwaves did not influence the total phenolic content, suggesting that polyphenols are strongly linked to starch chains, supporting the effect of cavitation, and ultrasound and microwave irradiation, respectively. Similarly, the results of antioxidant activity indicated that ultrasound and microwave significantly reduced (*p* < 0.05) compared to the conventionally extracted sample; this effect may be associated with localized thermal degradation and the generation of hydroxyl radicals induced by bubble formation during microwave and ultrasound treatments. During cavitation, the presence of these hydroxyl radicals in water can lead to the oxidation of certain polar compounds [32].

### 2.4. Technological Properties

#### 2.4.1. Water-Holding Capacity (WHC)

The water absorption capacity determines the weight of water that can be retained per unit weight of dry starch. The water-retention capacity (WHC) was expressed in g per 100 g of starch. The WHC ranged between 80.48 and 90.05 g/100 g of starch, as shown in Table 2. The WHC values for the MKS-UAE and MKS-MAE starches were significantly higher (*p* < 0.05) than for MKS-WMP starch. The increase in WHC in the sonicated sample could be explained by the cavitation-driven decomposition of starch molecules into small-sized granules, exhibiting enhanced specific exposed granule areas compared to those of conventionally extracted starch molecules [33]. Similarly, microwave-assisted extraction contributes to the increase in the specific net energy absorbed by starch, favoring the alteration of the morphological (crack formation) and molecular structure of the starch granules, followed by the entry of water into the interior regions. During microwave treatment, the removal of moisture causes severe tension between crystals, leading to transformation of starch nature from crystalline to amorphous and allowing unhindered reaction with water, which is represented as an increase in WHC [29]. These results are in agreement with the findings of Falsafi et al. [34] for sonicated oat samples that demonstrated a significant increase (*p* < 0.05) in WHC when compared to the non-sonicated sample, and the results obtained by Gani et al. [35] for starch from lotus stem harvested from Dal Lake of Jammu and Kashmir, India, with microwave application.

#### 2.4.2. Oil-Holding Capacity (OHC)

OHC is directly related to flavor retention and mouthfeel, making it an important parameter to consider. The OHC of MKS-WMP starch was 76.43 g/100 g of starch, which was reduced to 70.58 g/100 g after microwave-assisted extraction. This phenomenon may be attributed to a reduction in the number of hydrophobic sites or an increased tendency of starch to resist interaction with oil during treatment, as reported by Nadir et al. [36] in potato starch with microwave treatment. In contrast, ultrasound-assisted extraction caused an increase in OHC to 83.23 g/100 g; this increase is mainly due to the destruction of the starch structure by the cavitation effect, leading to greater capillary trapping of molecules of oil in the empty fraction and the filling of interstitial spaces between the starch granules. These findings are in agreement with those reported for potato and yam starch in studies where the impact of sonication on the aggregation structure and physicochemical characteristics was evaluated [37,38]. These findings indicate that ultrasound-assisted extraction enhances the emulsifying properties of MKS, broadening its potential applications in various food formulations.

#### 2.4.3. Solubility and Swelling Power (SP)

The solubility and swelling capacity values were evaluated at temperatures of 25, 65, and 90 °C, as shown in Table 2. In all cases, the SP values increased gradually with increasing temperature, which can be attributed to the weakening of hydrogen bonds between intermolecular interactions at high temperatures, further aggravating the swelling of the samples. In the case of ultrasound-assisted extraction, there were only significant differences (*p* < 0.05) at a temperature of 90 °C, which showed a decrease in the SP value when compared to MKS-WMP and MKS-MAE, possibly due to depolymerization of starch molecules caused by ultrasound; this leads to the breakdown of the amylopectin chain. Additionally, at elevated temperatures, starch undergoes gelatinization, and the resulting gelatinized starch, along with the denatured protein matrix, can hinder water penetration into the starch structure [39]. Similar results were found in pea starch extracted with ultrasound assistance and lentil starch modified by ultrasound [24,40]. However, microwave-assisted extraction did not modify the SP values when compared to the conventionally obtained sample. These results are not in accordance with those reported by Palay [41], who pointed out that microwave treatment decreased the swelling capacity and was due to gelatinization by microwave irradiation and the absence of a continuous network of amylose chains.

The solubility percentage of mango seed starch increased markedly with increasing temperature from 25 to 90 °C, which may be due to increased leaching of amylose with increasing temperature (*p* < 0.05). The increase in MKS-MAE starch solubility may also be correlated with the variation induced by microwave irradiation in the starch structure. Throughout this process, the movement of polar molecules, along with unavoidable friction, dipole rotation, and molecular collisions, enhances the functional properties of starch, particularly its solubility. Results that coincide with ours were recently reported for starch isolated from lotus seed flour (*Nelumbo nucifera*) thermally treated with microwaves [42]. The present results corroborate those from previous reports on the influence of microwave treatment on the functional properties of starches and seed flours [42,43,44].

### 2.5. Rheological Properties

**Figure 2:**
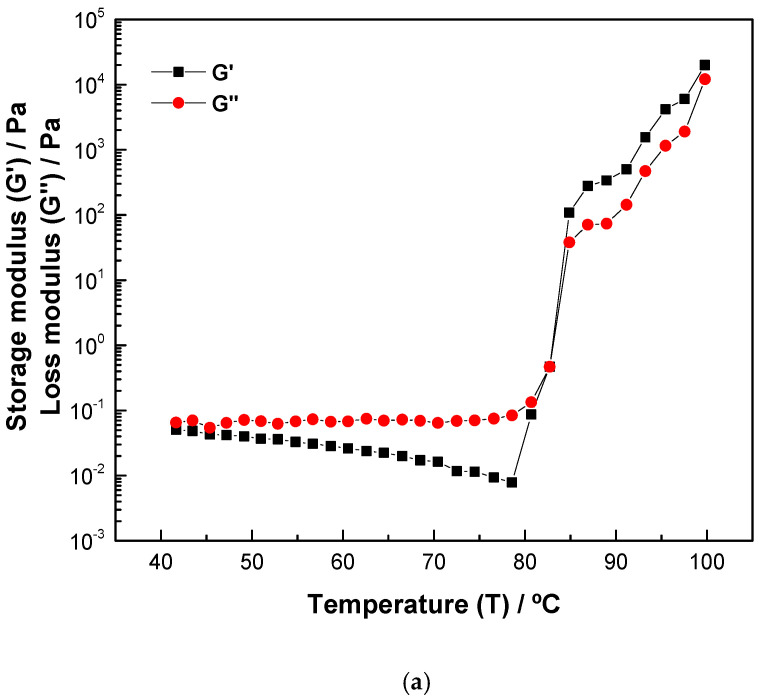

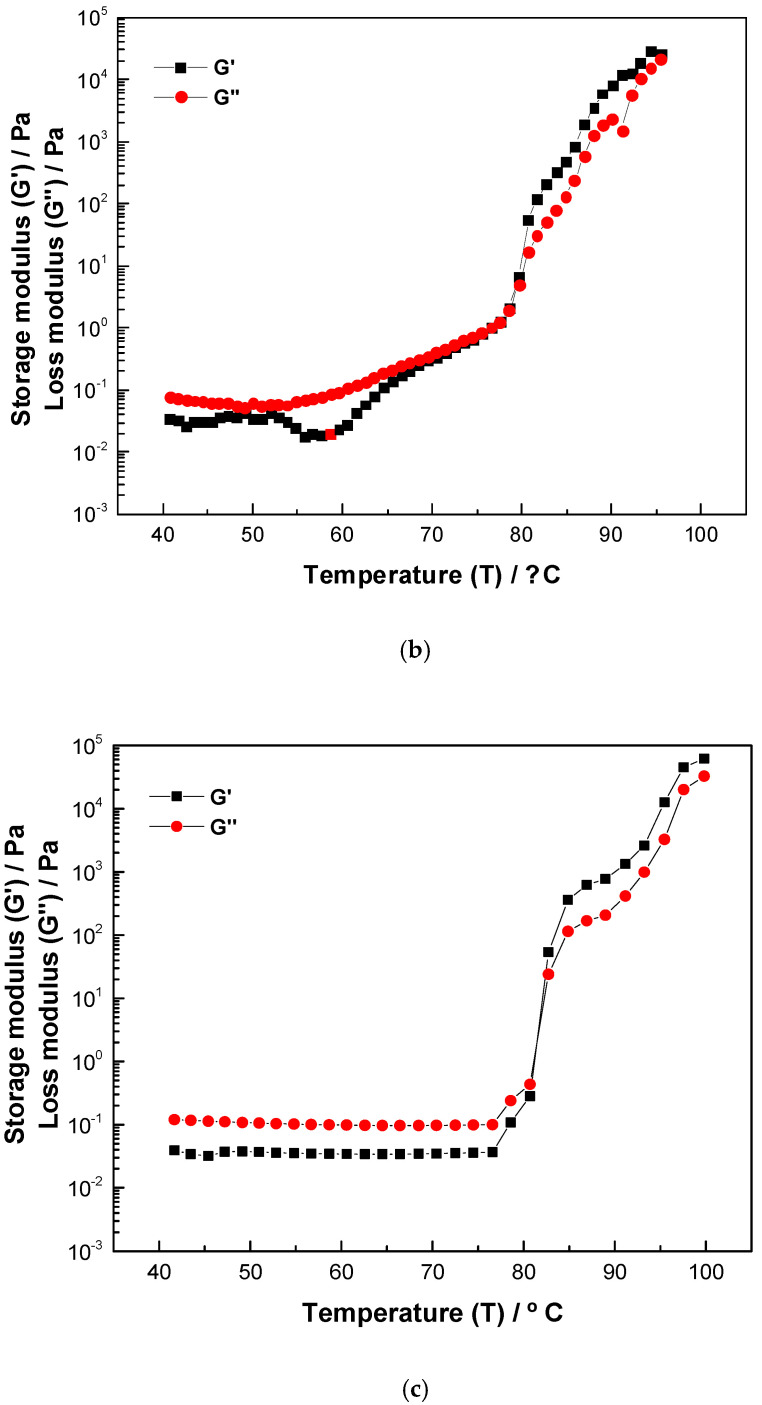
The storage (G′) and loss (G″) moduli of mango kernel starch by (**a**) wet-milling process (MKS-WMP), (**b**) ultrasound-assisted extraction (MKS-UAE), and (**c**) microwave-assisted extraction (MKS-MAE).

The gelatinization, defined as the intersection corresponding to the intersection of G′ (storage modulus) and G″ (loss modulus) [45] of samples, is shown in Figure 2. It was observed that, at the beginning of heating, within the range from 40 to 75 °C, the values of G′ were lower than those of G″ for all samples and were basically stable. After 75 °C, G′ and G” increased sharply. As the temperature increased, the two modules crossed (G′ = G″), indicating the start temperature of gelatinization of the studied starches.

The gelatinization temperature values measured during the rheological tests were 82.68, 77.52, and 81.70 °C for the MKS-UAE, MKS-MAE, and MKS-WMP samples, respectively. As the temperature increased, starch granules absorbed water, causing them to swell, while amylose was released and interconnected to create a network structure. However, further heating resulted in the melting, fragmentation, and breakdown of the starch granules, along with a weakening of the intermolecular interactions, so the G′ values were higher than those of G″, showing a different trend than the previous one. Similar behaviors have been found in different starch suspensions, such as potato, cassava [46], and Peruvian carrot starch peruana [47]. The gelatinization temperature of MKS-MAE starch was lower and presented significant differences from the rest of the samples (*p* > 0.05), This effect may be attributed to microwave irradiation, which significantly disrupted both internal and intermolecular hydrogen bonds within the starch structure. As a result, starch chains became more exposed, leading to a reduction in amylopectin content. Consequently, water molecules could infiltrate the starch granules more efficiently, facilitating gelatinization and lowering the gelatinization temperature [48]. Similarly, MKS-UAE starch maintained comparable gelatinization characteristics to the conventional sample (MKS-WMP), suggesting that ultrasound treatment did not significantly alter the overall crystalline structure or energy required for gelatinization. However, structural modifications observed in SEM images—such as surface fissures—could still enhance hydration and influence functional behavior in specific applications.

From an industrial perspective, the lower gelatinization temperature of MKS-MAE starch is advantageous for applications in thermally sensitive food products, such as instant puddings, sauces, or powdered beverages, for which rapid solubilization at lower temperatures is desired [49].

### 2.6. Pasting Properties

**Figure 3:**
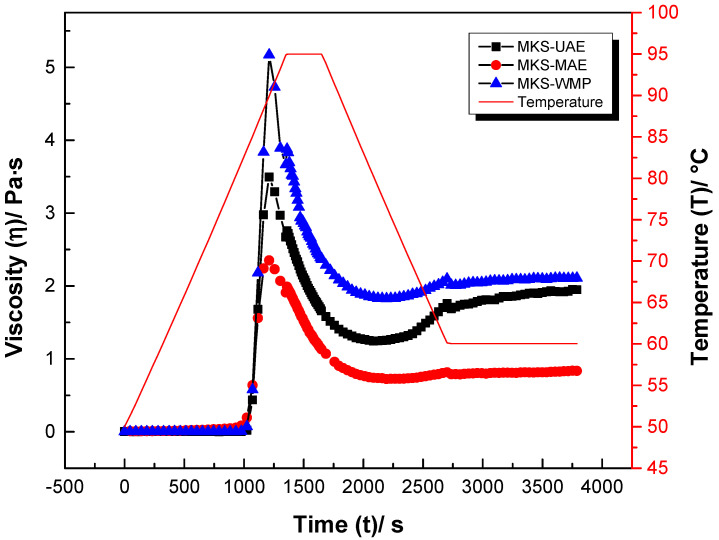
Pasting properties of mango kernel starch by wet-milling process (MKS-WMP), ultrasound-assisted extraction (MKS-UAE), and microwave-assisted extraction (MKS-MAE).

The profiles of the bonding property curves are shown in Figure 3, and the bonding parameters of the mango seed starches extracted with the assistance of ultrasound, with the assistance of microwaves, and conventionally are summarized in Table 3.

The PT of the MKS-WMP sample was 83.80 °C, higher than that of samples extracted with ultrasound and microwaves. Additionally, ultrasound and microwave treatments may modify the crystalline structure of starch granules, disrupt macromolecular chains, and decrease the granules’ rigidity and structural integrity, ultimately leading to a reduction in maximum viscosity [50]. The lower viscosity could be attributed to the partial breakdown of the integrity of the starch granules, which in turn led to a greater penetration of water for hydration, results that agree with the results obtained for the water-retention capacity of the samples MKS-UAE and MKS-MAE. Furthermore, Studies have indicated that the mechanical energy generated by ultrasound and cavitation can fragment long molecular chains and weaken the interaction forces between starch granules, ultimately resulting in a decrease in the peak viscosity [51].

The breakdown viscosity of the MKS-UAE, MKS-MAE, and MKS-WMP samples was found to be 2.26, 1.63, and 3.34 Pa·s, respectively. Consequently, the MKS-MAE sample exhibited the highest resistance to shear thinning under thermal processing, likely due to the degradation of glycosidic bonds induced by the vibration of polar molecules during microwave treatment. A similar behavior has been reported for chestnut starch subjected to microwave processing [52]. The low setback values reflect the stability of starch against deterioration and aging. In the present study, the setback viscosity values decreased significantly (*p* < 0.05) with the application of ultrasound and microwaves, and this effect may be attributed to the disruption of amylose rearrangement caused by ultrasonic treatment; microwave processing also contributed positively to this modification [53]. 

### 2.7. Scanning Electron Microscopy (SEM)

**Figure 4:**
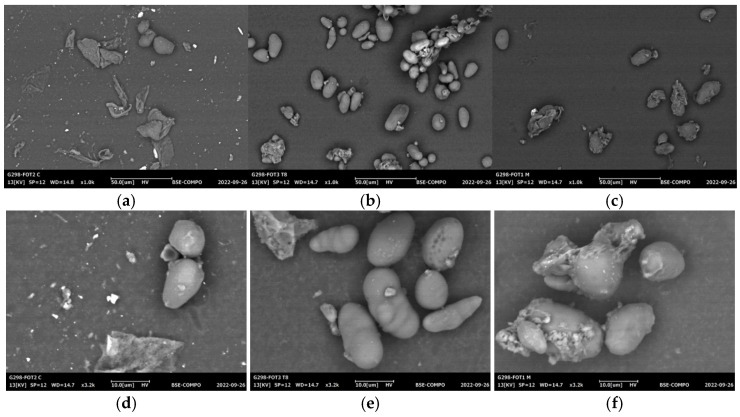
SEM micrographs of mango kernel starch by (**a**) wet-milling process (MKS-WMP), (**b**) ultrasound-assisted extraction (MKS-UAE), and (**c**) microwave-assisted extraction (MKS-MAE) at 1000X; and (**d**) MKS-WMP, (**e**) MKS-UAE, and (**f**) MKS-MAE at 3200X.

SEM micrographs of sample starches at 1000× and 3200× magnification are presented in Figure 4a–f. The extraction method influenced the surface morphology and granule integrity. In general, both UAE and MAE treatments led to a reduction in granule size compared to the conventional method. Also, MKS-WMP exhibits a distinct angular shape with an irregular surface (Figure 4a,d), and some granules show smooth surfaces, indicating partial preservation of native morphology, whereas MKS-UAE (Figure 4b,e) has an oval shape with agglomerate, a smooth surface, and small fissure depressions. These features are associated with the selective disruption of the amorphous regions of starch granules by ultrasound cavitation [54], which induces erosion and physical disintegration without completely collapsing the granule structure [55]. MKS-MAE (Figure 4c,f) presents irregular oval granules with more pronounced cracks, fissures, and surface scratches. The intense molecular movement induced by microwave energy may cause thermal stress, localized overheating, and fragmentation, resulting in granule deformation and structural collapse [26,56]. The higher degree of surface disruption observed in MAE samples could explain their higher solubility and water-holding capacity, as more porous structures allow for greater water penetration and starch chain mobility.

These microstructural differences are consistent with the technological properties discussed earlier. The disrupted surfaces observed in UAE and MAE starches may enhance their interaction with water and oil, influencing swelling, emulsification, and viscosity behavior—key attributes in food-formulation applications.

## 3. Conclusions

Starch extraction by UAE and MAE exhibited a significantly high extraction yield compared to the WMP method; in addition, the UAE and MAE technologies increased amylose content. The FTIR spectrum did not change, suggesting that ultrasound and microwave treatments exhibited solely physical effects on the starch samples; in addition, the UAE and MAE did not influence the total phenolic content. The technofunctional properties of WFC, OHC, solubility, and SP were improved through ultrasound- and microwave-assisted extraction, allowing for the expansion of these starches in different industrial applications. Extraction by MAE made it possible to accelerate the gelatinization process and reduce the gelatinization temperature. Ultrasound and microwave treatment decrease the size of mango kernel starch, improving the surface and shape. The results of this research indicated that UAE and MAE provide some novel characteristics to mango seed starch that contribute to increasing the applications of these starches in the food industry, such as instant powder type, meat industry, or stabilizers of microstructured products.

## 4. Materials and Methods

### 4.1. Materials

Anhydrous sodium carbonate (99.5% purity) and sodium bisulfite (99.5% purity) were purchased from Sigma-Aldrich (St. Louis, MO, USA). Ethanol (99.5% purity) was purchased from Panreac (Barcelona, Spain). Mango (*Mangifera indica*) var. Corazón fruits were harvested to commercial maturity.

### 4.2. Obtention of Mango Kernel Flour

In order to separate de kernel, the mango seed was washed and dried at 40 °C for 12 h in a hot air -tray dryer and milled to obtain a flour with a particle size less than 250 µm.

### 4.3. Wet-Milling Process of Mango Kernel Starch

The obtention of mango kernel starch by wet-milling process (MKS-WMP) was carried out following the methodology proposed by Kaur et al. [8], with some modifications. A suspension of 10 g of flour was made in 90 mL of aqueous sodium bisulfite solution (1%) and was stirred for 4 h at room temperature. Subsequently, the homogenization was carried out on an Ultra-Turrax (IKA T25, Staufen, Germany) at 650 rpm × 5 min in order to ensure that the raw material was completely solubilized. The mixture was filtered, and the cake was washed with distilled water. Then, the filtrate was centrifuged at 5000 rpm for 10 min, and the precipitate was dried at 40 °C until constant weight. The extraction yield of starch was calculated based on the initial dry weight amount of mango flour (mango kernel flour) and obtained starch (dry starch), as expressed in Equation (1).

(1) Yield (%)=Dry starch (g)Mango kernel flour(g)×100

### 4.4. Ultrasound-Assisted Extraction (UAE) of Mango Kernel Starch

The obtention of mango kernel starch by ultrasound-assisted extraction (MKS-UAE) was carried out following the procedure described by Mieles et al. [57]. The procedures for wet-milling process of mango kernel starch applying an ultrasound to the suspensions in solubilization step using an Ultrasonic Processor (SX Sonic, FS-1200N, Shanghai, China) with a 20 mm diameter probe that was submerged at a depth of 50 mm from the surface of the suspension, operating at 360 W for 10 min at 40 °C and constant frequency of 20 kHz, with pulse durations of 2 s on and 2 s off.

### 4.5. Microwave-Assisted Extraction (MAE) of Mango Kernel Starch

The obtention of mango kernel starch by microwave-assisted extraction (MKS-MAE) was carried out using the methodology described by Ramírez-Brewer et al. [58], with some modifications. The procedure for the wet-milling process of mango kernel starch involves applying microwaves on the suspensions in a solubilization step for a period of 90 s, using a microwave oven at a low–medium power of 360 W.

### 4.6. Determination of the Amylose and Amylopectin Content

The amylose content was determined through the iodine method, following the procedure described by Morrison and Laignelet [59].

### 4.7. Fourier Transform Infrared Spectroscopy (FTIR) Analysis

Fourier-transform infrared spectroscopy (FTIR) in a medium spectrum range was performed in order to identify the functional groups of starch, employing an FTIR spectrometer (Shimadzu model IR-Affinity, Kyoto, Japan). The samples were combined with KBr at a 1:100 (*w*/*w*) ratio and dried, and then their IR spectra were recorded within the 400–4000 cm^−1^ wavelength range, with a resolution of 4 cm^−1^, at room temperature. The intensity of the spectra was measured by recording the height of the absorbance bands relative to the baseline.

### 4.8. Determination of Total Phenolic Compounds (TPC) and Antioxidant Capacity

The total phenolic content of samples was determined using the Folin–Ciocâlteu method [60], and the results were expressed as mg gallic acid equivalents (GAE)/g starch. The antioxidant capacity was measured following the method described by Re et al. [61], using the ABTS free radical scavenging test. The results were expressed as µmol of Trolox (TEAC)/g of starch.

### 4.9. Technological Properties Analysis 

The water-holding capacity (WHC) and oil-holding capacity (OHC) of the samples were evaluated using the methodology outlined by Yamazaki [62], modified by Medcalf [63].

The solubility (weight of dried supernatant/weight of sample × 100) and swelling power (weight of wet sediment/weight of sample (100%-% solubility) × 100) of the starch samples were assessed following the methodology described by Jiang et al. [64].

### 4.10. Rheological Analysis 

Starch was mixed with distilled water in order to obtain suspensions at 15% *w*/*w*. The suspensions were subjected to rheological analysis, using the procedure established by López-Barraza et al. [65]: employ a controlled stress rheometer (Modular Advanced Rheometer System Haake Mars 60, Thermo-Scientific, Dreieich, Germany) equipped with a rough plate geometry (35 mm diameter and GAP diameter) to prevent wall slip effects.

Stress sweep: In order to identify the linear viscoelastic region (LVR), a stress amplitude sweep test of samples was performed within a range from 0.001 to 1000 Pa, at angular frequencies of 0.01, 0.1, and 1 Hz at 25 °C.

Temperature sweep: The temperature sweep was conducted within the LVR from 40 to 90 °C, using a heating rate of 5 °C/min at a constant frequency of 1 Hz.

Pasting properties: The temperature profile was determined following the methodology described by Mieles et al. [58]: briefly, heating ramps start from 50 to 95 °C at 5 °C/min, hold at 95 °C for 5 min, cool down from 95 to 60 °C at 5 °C/min, and finally kept at 60 °C for 5 min.

### 4.11. Microstructural Analysis

Microstructural analysis was performed by employing a scanning electron microscope (JSM-6390LV, JEOL, Ltd., Tokyo, Japan) operating at 5 keV. The samples were affixed to aluminum stubs using double-sided carbon tape and coated with a thin gold layer through ion sputtering under vacuum conditions.

### 4.12. Statistical Analysis

Data were analyzed with a unidirectional ANOVA, using Statgraphics software (version centurión XVI), in order to determine statistically significant differences (*p* < 0.05) between samples. The Least Significant Difference (LSD) test was used for the comparison of means. All tests were performed in triplicate.

## Figures and Tables

**Table 1:** Yields (g of dry starch/g of mango kernel flour × 100), amylose content, total phenolic content (TPC), antioxidant activity (AA) of mango kernel starch by wet-milling process (MKS-WMP), ultrasound-assisted extraction (MKS-UAE), and microwave-assisted extraction (MKS-MAE).

Sample Code	Yield %	Amylose Content g/100 g	TPC mg GAE/g	AA µMol Trolox/g
MKS-WMP	42.05 ± 2.58 ^b^	28.46 ± 0.93 ^b^	84.89 ± 1.55 ^a^	18.15 ± 1.10 ^b^
MKS-UAE	50.40 ± 2.26 ^a^	33.84 ± 1.47 ^a^	90.85 ± 3.08 ^a^	16.12 ± 1.50 ^a^
MKS-MAE	48.43 ± 1.41 ^a^	32.21 ± 1.58 ^a^	89.60 ± 4.11 ^a^	15.24 ± 0.56 ^a^

Data are the mean ± standard deviation. Different letters in the same columns express statistically significant differences (*p* < 0.05).

**Table 2:** Water-holding capacity (WHC), oil-holding capacity (OHC), solubility, and swelling power (SP) of mango kernel starch by wet-milling process (MKS-WMP), ultrasound-assisted extraction (MKS-UAE), and microwave-assisted extraction (MKS-MAE).

Sample Code	WHC g/100 g	OHC g/100 g	Solubility			Swelling Power		
			25 °C	65 °C	90 °C	25 °C	65 °C	90 °C
MKS-WMP	80.48 ± 2.41 ^b^	76.43 ± 1.63 ^b^	1.10 ± 0.07 ^c^	6.20 ± 0.15 ^c^	15.00 ± 0.58 ^b^	1.97 ± 0.09 ^b^	6.17 ± 0.04 ^a^	14.88 ± 0.47 ^b^
MKS-UAE	87.76 ± 2.01 ^a^	83.23 ± 1.83 ^a^	1.52 ± 0.04 ^a^	6.86 ± 0.08 ^a^	16.75 ± 0.30 ^a^	2.74 ± 0.32 ^a^	6.32 ± 1.02 ^a^	12.89 ± 0.85 ^a^
MKS-MAE	90.05 ± 2.54 ^a^	78.56 ± 1.32 ^a^	1.34 ± 0.05 ^b^	6.46 ± 0.12 ^b^	16.88 ± 0.38 ^a^	2.49 ± 0.46 ^ab^	6.45 ± 0.74 ^a^	15.72 ± 1.10 ^b^

Data are the mean ± standard deviation. Different letters in the same columns express statistically significant differences (*p* < 0.05).

**Table 3:** Pasting properties of mango kernel starch by wet-milling process (MKS-WMP), ultrasound-assisted extraction (MKS-UAE), and microwave-assisted extraction (MKS-MAE). PT, pasting temperature; PV, peak viscosity; TV, trough viscosity; BD, breakdown viscosity; FV, final viscosity; SB, setback viscosity.

Sample Code	PT °C	PV Pa·s	FV Pa·s	TV Pa·s	BV Pa·s	SB Pa·s
MKS-WMP	83.80 ± 1.67 ^b^	5.17 ± 0.25 ^c^	2.11 ± 0.10 ^c^	1.83 ± 0.09 ^c^	3.34 ± 0.16 ^c^	3.06 ± 0.15 ^b^
MKS-UAE	82.84 ± 1.47 ^ab^	3.49 ± 0.19 ^a^	1.94 ± 0.07 ^a^	1.23 ± 0.08 ^a^	2.26 ± 0.15 ^a^	1.54 ± 0.05 ^a^
MKS-MAE	79.85 ± 1.41 ^a^	2.35 ± 0.12 ^b^	0.83 ± 0.05 ^b^	0.72 ± 0.09 ^b^	1.63 ± 0.11 ^b^	1.52 ± 0.10 ^a^

Data are the mean ± standard deviation. Different letters in the same columns express statistically significant differences (*p* < 0.05).

## Data Availability

The original contributions presented in this study are included in the article. Further inquiries can be directed to the corresponding author.
